# First characterization and risk assessment of microplastics in the endangered Indus River dolphin (*Platanista minor*): Implications for conservation strategies

**DOI:** 10.1371/journal.pone.0330253

**Published:** 2025-09-24

**Authors:** Ahsaan Ali, Christian Sonne, Mehmood Aslam, Imran Ali, Sajjad Hussain, Abdul Qadir, Shazia Sukhera, Hassan Ali, Sajid Rashid Ahmad, Guang Yang

**Affiliations:** 1 Jiangsu Key Laboratory for Biodiversity and Biotechnology, College of Life Sciences, Nanjing Normal University, Nanjing, China; 2 Department of Ecoscience, Faculty of Technical Sciences, Aarhus University, Roskilde, Denmark; 3 MOE Key Laboratory of Pollution Processes and Environmental Criteria, College of Environmental Science and Engineering, Nankai University, Tianjin, China; 4 KEO International Consultants, United Kingdom; 5 Punjab Wildlife and Parks Department, Lahore, Pakistan; 6 College of Earth and Environmental Sciences (CEES), University of the Punjab, Lahore, Pakistan; 7 Institute of Molecular Biology and Biotechnology (IMBB), University of Lahore, Lahore, Pakistan; King Fahd University of Petroleum & Minerals, SAUDI ARABIA

## Abstract

Microplastic (MP) pollution has become a global environmental concern due to its ubiquitous presence and potential threats to ecosystems. Cetaceans, as top predators, have served as sentinel species for monitoring ecosystem changes and as flagship species for establishing environmental conservation strategies. Here, we investigated MPs for the first time in the complete gastrointestinal tract of five individuals of an endangered Indus River dolphins (*Platanista minor*) stranded along the Indus River from 2019−2022. MPs were detected from all examined specimens with an average of 286.4 ± 109.1 MPs per individual, indicating the potential pathways for the accumulation of MPs due to prey consumption and unintentional ingestion during food uptake in Indus River dolphin. The properties of MPs demonstrated that the most prevalent shapes were fibers, with sizes mostly ranging from 5 mm-300µm. Polyethylene terephthalate (PET) was the predominant composition identified via FT-IR spectroscopy. Moreover, the prevalence of MPs observed in the small intestine was higher than in other parts due to length and structure. This is the first study to highlight the ecological risks posed by MP polymers through a polymer risk assessment (H) showing medium (Level III) to high risks (Level IV) to Indus River dolphins. This study represents the first baseline assessment of MPs pollution caused by anthropogenic activities and offers valuable insights for the conservation of this endangered freshwater species. Our results emphasize the need for further ecotoxicological studies to better understand the potential impacts of MPs in this endangered species.

## Introduction

Plastic pollution has been a growing global concern since the 1970s, as its affordable, accessible, and versatile nature has driven excessive production and accumulation in aquatic ecosystems worldwide [1–3]. Consequently, it is estimated that the accumulated amount of plastic dumped into the ocean since 2010 accounts for 4.8 to 12.7 million tons [4]. Macro plastic waste is a widespread and pervasive issue causing ecological threats. Its extensive distribution leads to habitat degradation, water quality decline, disruption of planktonic ecosystems, food chain imbalances, reduced population growth and reproduction rates [5–7]. Plastic exists in various sizes divided into macro-plastics (>200mm), meso-plastics (5–200 mm), and microplastics (<5mm, hereinafter MPs) [8]. While size-based classification is useful, MPs are further categorized by origin into primary and secondary types [9]. Primary MPs are produced in the form of microbeads, and microfibers, and are present in personal care products, cosmetics, and air blasting, and are used as raw material in different industrial processes [10,11]. Secondary MPs are formed by the degradation of plastic materials that experience physiochemical weathering when they are released into the environment, e.g., food packaging and fishing nets. The environmental process of degradation includes photo-oxidation, mechanical abrasion, biodegradation, and UV radiation [12,13]. In aquatic ecosystems, MPs pose significant threats due to their ability to transport antibiotic resistance genes (ARGs), toxic plasticizers, and pathogenic microorganisms [14,15].

MP ingestion frequently occurs in aquatic organisms including dolphins, fish and crustaceans primarily through trophic transfer which may reduce food intake, energy storage, growth, and reproductive capacity [16–19]. MP ingestion can lead to toxic effects as it accumulates environmental contaminants including plasticizers, heavy metals, and persistent organic pollutants (POPs) [20–22]. The ingestion of MPs by low trophic organisms can lead to the transfer of potentially toxic compounds through the aquatic food web, causing adverse effects such as metabolic disorders, neurotoxicity, immunotoxicity, and developmental toxicity [14,23,24]. MPs also have the potential to absorb high levels of POPs from the environment, which can lead to organ dysfunction during co-uptake [25–28]. Significant MP contamination has been reported in the Indus River basin. Previous study from the Swat River reported 192 items/L in surface water and 182 items/kg in sediments [29]. In the Ravi River, MP levels exceeded 2,000 items/L in water and 2,300 particles/20g of dry sediment [30,31]. MPs were also detected in potential IRD prey species at Panjnad barrage, including *Labeo rohita*, *Wallago attu*, and *Cirrhinus mrigala*, with PET, PE, PP, and PVC as dominant polymers [32]. These findings highlight widespread contamination and possible trophic transfer to Indus River dolphins. Assessing MPs exposure in endangered freshwater cetaceans is critical for evaluating ecological and conservation impacts.

The biodiversity of freshwater environments is declining at a higher rate compared to terrestrial ecosystems [33,34]. The Indus River dolphin (IRD, *Platanista minor*) is an endangered freshwater cetacean endemic to the Indus River system in Pakistan and a few individuals in India, listed as endangered by IUCN Red List of Threatened Species [35–37]. The IRD is distributed within 900 km of the Chashma to Kotri barrage and persists in three sub-populations and a small population exists downstream of Sukkur barrage in the Indus River’s mainstream, each separated by irrigation barrages [38]. As top predators, IRD serves as sentinel species for environmental changes and a flagship species for aquatic conservation [36]. Cetaceans, with their long lifespans and high-trophic-level diets, are particularly vulnerable to industrial pollution including MPs [39,40]. This makes them valuable bioindicators for assessing potential human health impacts in the Indus River ecosystem [12,41,42].

Here we present the first characterization and risk assessment of MPs in the endangered IRDs. Given previous studies identifying high pollution levels in their habitat, it is expected that IRDs are highly exposed to MPs pollution. The objective of this study is to evaluate, quantify, characterize, and analyze MPs in the complete gastrointestinal tract (GIT) of stranded IRDs in relation to body length. In addition, our study revealed the potential ecological risks of MPs in IRDs by calculating the polymer risk indexes (H).

## Materials and methods

### Study area

The Indus River, one of the largest rivers in Pakistan, originates from Tibetan Plateau, enters Pakistan in the northern region of Gilgit-Baltistan, flowing southward where it flows down across the country before emptying into the Arabian Sea. The IRD is native to the Indus River system in Pakistan and ranks among the most endangered cetaceans worldwide [43,44]. Currently, the IRD population is distributed into the three sub-populations i) Chashma to Taunsa, ii) Taunsa to Guddu, iii) Guddu to Sukkur, and a small population persists downstream of Sukkur barrage. This distribution is shaped by six irrigation barrages on the Indus River in Pakistan [45]. The study area for this research was selected between the Chashma to Taunsa barrage (C-T) and Taunsa to Guddu barrage (T-G) encompassing almost 538 km. From 2019-2022 (mid-January to mid-April), we conducted collaborative boat-based surveys with the Punjab Wildlife Department at an average speed of 10–15 km/h, using binoculars and direct visual observations to monitor stranded dolphins under official permission (Vide letter No. 1965/DDW/DGK/2018 dated 8 Oct 2018). All surveys were strictly non-invasive.

### Necropsy and sample collection

Stranded IRD carcasses were contributed for research by the Punjab Wildlife Department. Preliminary examinations with morphometric measurement including total body length (cm), anterior and posterior length, flippers and fluke’s length, girth measurement, sex determination, body weight, stranding location, decomposition state, any evidence of fisheries and human interaction. A careful external physical examination revealed a set of bruises and scars on the antero-lateral sides of three out of five stranded IRD carcasses. Basic statistics on each individual including age, sex and morphometrics is found in Table 1. To ascertain the age of carcasses, we measured standard length and counted the growth layer group (GLGs) of dentin, following the method used for striped dolphins and common bottlenose dolphins [46,47]. The carcasses were subsequently transferred to CEES, University of the Punjab, Lahore and kept at −20°C until necropsy.

**Table 1:** Basic information of the stranded IRDs is included in this study.

Sample ID	Sex	Length (cm)	Weight (kg)	Body condition	Location
IRD01	Female	102	18.92	Good	32.27840N, 71.31643E
IRD02	Female	118	21.3	Good	30.97398N, 70.87968E
IRD03	Male	116	13.87	Normal	30.46943N, 70.86849E
IRD04	Male	149	25.66	Very good	30.10510N, 70.79919E
IRD05	Female	181	33.12	Very good	28.92247N, 70.54270E

Necropsies were performed by an expert team comprising of medical anatomist, zoologists and toxicologists, following the standard procedures described by Geraci et al. [48] and Plön et al. [49]. All necropsies were carried out using sterile and stainless-steel material including necropsy table, scissors, trays, surgical scalpels with blades, and bone cutter. After necropsy, the weight of complete GIT (stomach, small and large intestine) and vital organs (liver, heart, kidneys, and lungs) were noted. The length of small and large intestines of each individual were particularly measured. The protocol for the collection of gut contents followed the procedures described for detection of MPs in Chinese white dolphins (*Sousa Chinesis*) [7]. The complete gut contents of each dolphin were extracted and subsequently preserved at a temperature of −20°C prior to further investigations. The protocols for the collection of samples were modified to include the complete GIT of each individual, while the remaining parts of the dolphins were wrapped and stored at −20°C for further future studies. Each GIT was weighed and measured before processing. For the convenience of sampling, each GIT was divided into four parts, i.e., 1) esophagus + fundic stomach, 2) main stomach + pyloric chamber. 3) small intestine and 4) large intestine. The procedures were carried out systematically in adherence to ethical standards and obligations of Nanjing Normal University, China.

### Gut content extraction

The procedure for washing the GIT followed the method of Lusher et al. [50,51] with modifications. The gut contents were taken out from the freezer and allowed thawing in 24h at room temperature in separate stainless-steel trays. Each part of the gut content was then dissected using stainless-steel surgical scissors and inverted in a steel tray. The stomach contents were passed through a series of four metal sieves with gradually decreasing mesh sizes (5 mm, 300µm, 150µm, and 50µm) to remove prey remains and prevent the sieves from getting blocked. To facilitate future research on diet, stomach contents including crab remains, fish bones, and large prey items including fish were carefully rinsed with filtered water and stored at −20°C for future studies of trophic MP transfer. Each part of the stomachs and intestines were washed with pre-filtered water using sieves (300µm, 150µm, and 50µm), and the wash solution was then transferred into separate glass beakers, covered with aluminum foil paper and tagged with their names (esophagus + fundic stomach, main stomach + pyloric chamber, small intestine, and large intestine).

### Digestion and isolation of MPs

The resulting material obtained from washing three sieve fractions were subjected to chemical digestion to extract MPs items. The washed gut content was digested using a 10% potassium hydroxide (KOH) solution (Sigma Aldrich, CAS: 1310-58-3), and filtered through a cellulose fiber filter with a pore size of 0.45µm [7,52]. The volume of KOH solution was three times greater than the sample was used. Samples were covered with aluminum foil paper to avoid contamination and placed in an oven at 60°C for at least 72h [53,54]. The solvent was thoroughly stirred several times throughout the procedure using a glass rod to achieve a homogeneous solution. The digestion period could last up to seven days, depending on the quantity of organic matter in the sample. After digestion, the sample solution was filtered again using a Buchner filter flask equipped with a vacuum pump. The filter-paper containing MPs was transferred to petri dishes, covered with aluminum foil, and dried at room temperature for 24h for detailed MPs investigations.

### Identification and characterization of MPs

Finally, the dried filter papers containing MPs were examined under a stereomicroscope (model NZ 1903S, Euromex Microscope), and photographs were taken using a mounted Euromex Microscopic Camera (CMEX 18 Pro, 18 MP, CMOS Sensor 1/2.3”) attached to a microscope. Visual examination and hot needle test, when necessary were performed to confirm the suspected MPs on the filters. Identified MPs were quantified and characterized based on shape (fibers, sheets/films, fragments, beads, and foam), colors (transparent, blue, black/grey, red, green, purple, pink, and white), and size ranges (5 mm-300µm, 300µm-150µm, and 150µm-50µm) [55]. The total MP count per individual in each part of GIT and their mean ± SD are presented in Table 2. Following the quantification and classification, polymer composition of MPs were determined using ATR FT-IR (Attenuated Total Reflection Fourier Transformed Infrared Spectroscopy) with an Agilent Technology Cary 630 spectrometer with a success ratio of 85% [56]. To prevent contamination and ensure accurate results, the ATR diamond and base were cleaned with ethanol before and after the process. The polymer verification was carried out on a spectral range of 4000−650 cm^−1^ and at a resolution of 4 cm^−1^. We used the Agilent Micro-lab native software of the spectrometer to analyze each spectrum and its built-in library to confirm the polymer type of MPs.

**Table 2:** MPs statistics for combined GIT compartments of dead carcasses of IRDs reported in the present study.

GIT Compartments	IRD01	IRD02	IRD03	IRD04	IRD05
Esophagus + Fundic Stomach	81	89	64	32	48
Main Stomach + Pyloric Chamber	98	104	50	71	64
Small Intestine	102	174	48	57	62
Large Intestine	96	62	22	59	49
Total	377	429	184	219	223
mean ± SD	94.3 ± 9.2	107.3 ± 47.8	46 ± 17.5	54.8 ± 16.4	55.8 ± 8.4

### Ecological risk assessment of MPs

MPs constitute a potential risk to aquatic organisms [57]. The polymer risk index (H) was used to evaluate the ecological risks of MPs. Previous studies indicate that the accumulation of MPs in aquatic organisms is mostly found in guts, therefore H values were calculated based on total MPs in the gut [58,59]. Due to variations in MP polymers toxicity, polymer risk index (H), were derived using hazard scores described by Lithner et al. [60]. The calculation of H values was demonstrated as follows.


$$H_{i}=\sum \left(\frac{P_{ji}}{MP_{i}}\times S_{j}\right)$$

where $H_{i}$ indicates the polymer risk index as a consequence of MPs, $P_{ji}$ refers to the number of polymers identified in sample i, $MP_{i}$ represents the abundance of MPs in sample i, and $S_{j}$ refers to identified polymer score assigned by Lithner et al. [60]. The polymer risk values and corresponding ecological risk categories of H were presented in Table 4, S5 and S6 Tables respectively [59].

**Table 4:** Ecological risk assessment (H) of MP polymers of the IRDs in the present study.

Sample ID	PET	PPS	PES	PVC	PU	PE	H^a^	Risk level^a^
IRD01	0.36	19.03	0.05	26.53	78.34	0.00	124	IV
IRD02	0.43	27.18	0.04	0.00	0.00	0.08	28	III
IRD03	0.57	14.63	0.07	0.00	40.13	0.00	55	III
IRD04	0.51	24.58	0.07	182.67	0.00	0.00	208	IV
IRD05	0.66	40.22	0.07	89.70	66.22	0.00	197	IV
Criteria of hazards	H^a^	0-1	1-10	10-100	100-1000	>1000		
	Risk level^a^	I	II	III	IV	V		
	Assessment declaration^a^	Very low	Low	Medium	High	Extremely high		

Note: The color of cells represents the degree of hazards such as level I (green), level II (blue), level III (yellow), level IV (orange) and level V (red).

^a^ Li et al. [60].

### Contamination control

To ensure the reliability of MP analysis, strict precautions were implemented to minimize contamination throughout all stages of sample handling and experimental procedures. All the laboratory surfaces and equipment were cleaned with 70% ethanol to avoid contamination. During the whole process, 100% cotton lab coats, face mask and nitrile gloves were worn. To confirm any procedural contamination during the process, three procedural blanks of Milli-Q water were placed and processed along each sample of GIT. Procedural blanks were processed alongside the samples during dissection, washing, sieving, digestion, filtration, and FTIR analysis to observe any potential background contamination. No significant contamination was observed in the blanks, confirming the integrity of the procedures. Reagents were stored in glass bottles to reduce the risk of introducing plastic related contaminants. Analytical grade KOH was used and prepared with Milli-Q water and used after filtration. Only stainless-steel and glass material was used in this study and this material was rinsed thoroughly with filtered water in advance. The samples were covered with aluminum foil when they were not in use to avoid any contamination.

### Ethical statement

The animal care and use committee of Nanjing Normal University approved all procedures and considerations for sampling and utilization of toxicological data. This complies with the National Animal Care Standard (GB 14925−2010). Carcasses were contributed by the Punjab Wildlife Department, Pakistan for the purpose of research only. We focused on reducing animal suffering at every stage of the process.

### Inclusivity in global research

Additional information regarding the ethical, cultural, and scientific considerations specific to inclusivity in global research is included in the Supporting Information (S1 Checklist).

### Statistical analysis

Statistical analyses were performed using SPSS v27.0 (IBM Corp., Armonk, NY, USA). An independent t-test was performed for the comparison of the shape and size of MPs between sexes and to ascertain the abundance of MPs in the different GIT compartments. A Kruskal-Wallis’s test was performed to evaluate the differences in color distribution between the specimens, and to determine whether there was a correlation between the length of the specimens and the abundance of MPs, the Spearman correlation test was performed. Statistical results were visualized using OriginLab (OriginPro 2024) and the study area map was created using ArcGIS Pro 3.2 (Esri).

## Results

### Abundance of MPs in GIT

**Fig 1:**
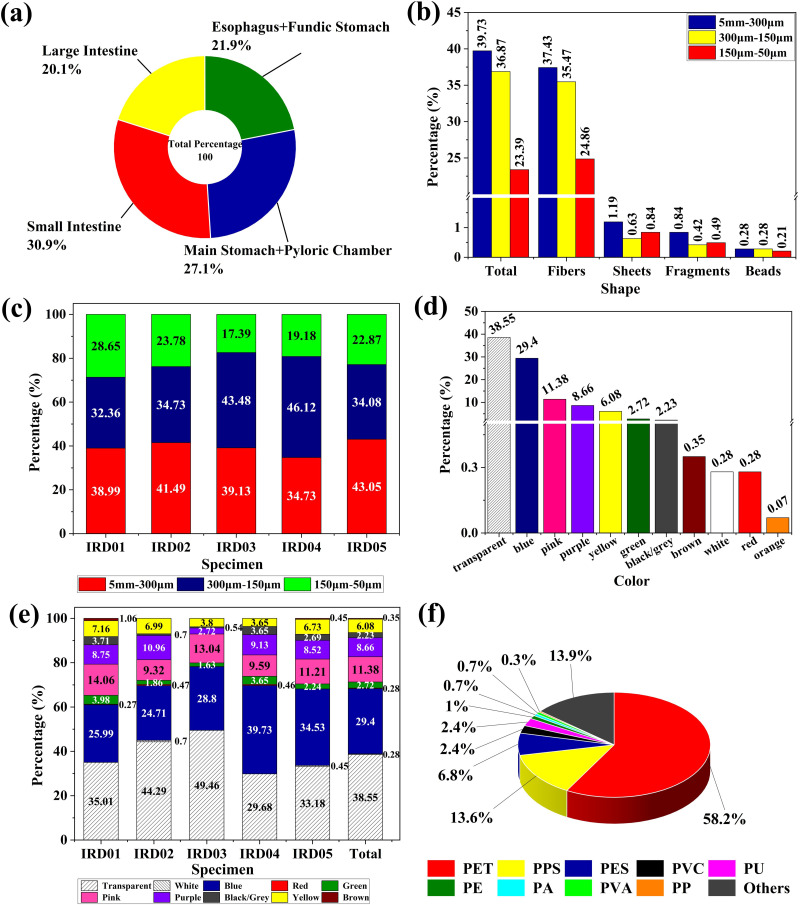
MP characteristics observed in the gut of all examined IRDs. (a) Total contribution of MPs distributed by GIT sections across all individuals (b) distribution of MP shapes categorized by size ranges (c) specimen-wise distribution by size ranges (d) overall color distribution across all individuals (e) specimen-wise color distribution across all individuals (f) polymer types identified across all GIT samples.

The results confirmed MPs in all four gut compartments of the dolphins (MP occurrence was 100%). The abundance of MPs ranged from 184 to 429 and on average 286.4 ± 109.1 MPs (mean ± SD) per dolphin (Table 2). In addition, the GIT of each dolphin sample was divided into four compartments, i.e., esophagus + fundic stomach, main stomach + pyloric chamber, small intestine, and large intestine, with MPs detected at a range of 32−89, 50−104, 57−174, and 22−96 items, respectively. Moreover, the highest abundance of MPs was positively correlated with the length of small intestine (Fig 1a). No significant differences were observed between different GIT compartments (t = −1.749, n = 5, *p = 0.179*). Similarly, the number of ingested MPs did not show any significant relationship with body length (R = −0.10, n = 5, *p = 0.873*).

### Shape and size characterization of MPs in GIT

Five MP morphologies were identified in the gut contents. Fiber possessed a significant prevalence throughout the dolphin individuals with a mean concentration of 271.4 ± 101.6 followed by sheets (6.6 ± 6.84), fragments (6.0 ± 4.53), beads (2.2 ± 3.35), and foam (0.2 ± 0.45) (S1 Table). There were no significant differences in shape among the sampled individuals as (t = −1.669, n = 5, *p = 0.194*). On the other hand, the size fractions showed that MPs of size 5 mm-300µm, 300µm-150µm, and 150µm-50µm were found with a mean concentration of 113.8 ± 46.68, 105.6 ± 30.44, and 67.0 ± 35.4, respectively (Fig 1b; Table 3 and S2 Table). The results of size fraction analysis revealed that the number of MPs in the fraction 5 mm-300µm was highest of all sieves. No significant differences were found in MP size within the GIT compartments and between the specimens (t = −0.627, n = 5, *p = 0.575*) (Fig 1b and 1c).

**Table 3:** Sieve-wise distribution of MPs from all individuals.

Sample ID	5mm-300µm	300 µm-150µm	150µm-50µm	Total
IRD01	147	122	108	377
IRD02	178	149	102	429
IRD03	72	80	32	184
IRD04	76	101	42	219
IRD05	96	76	51	223
Total	569	528	335	1432
Mean	113.8	105.6	67.0	286.4
SD	46.68	30.44	35.4	109.1
% MPs	39.73	36.87	23.39	100

### Color and composition of MPs in GIT

The MPs were identified in different colors from all dolphin individuals (transparent/clear, blue, pink, purple, yellow, green, black/grey, brown, white). In general, the dominant percentage of MPs were transparent/clear and blue which were 110.4 ± 51.4 and 84.2 ± 20.6 of the total MPs detected from different parts of all specimens, respectively (Fig 1d, S3 Table). Color-wise distribution of MPs concerning each specimen is presented in Fig 1e. There were no significant differences in MP color that were detected between the sexes (p > 0.05 or *p = 0.406*).

**Fig 2:**
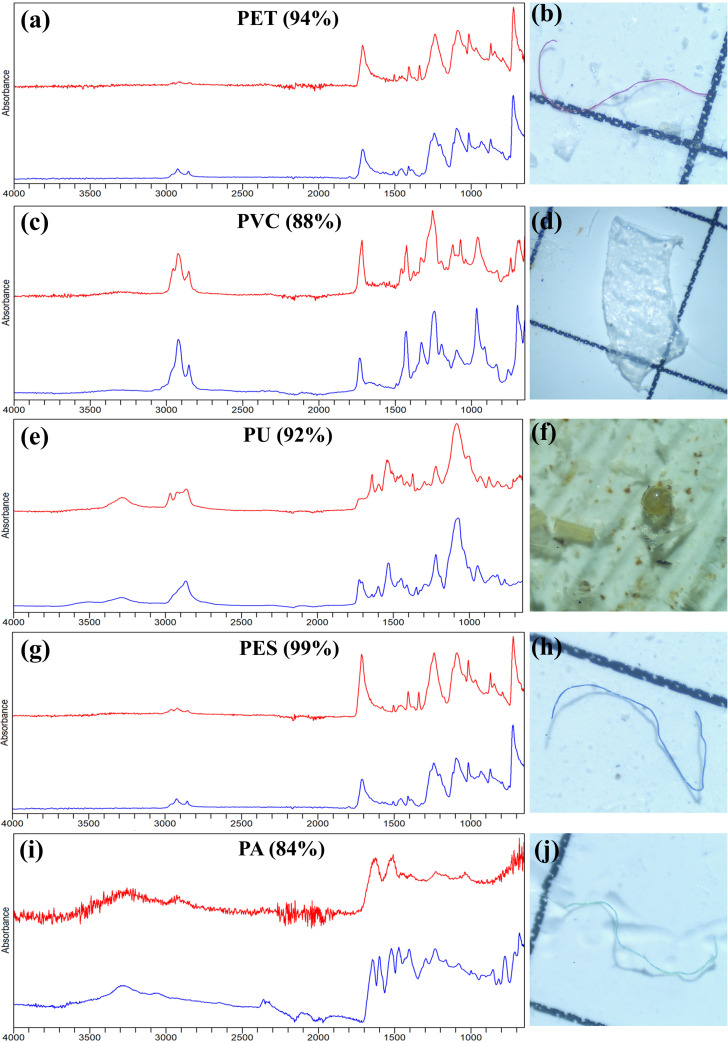
a,c,e,g,i shows FTIR spectra of MP items, while b,d,f,h,j displays corresponding photographs of the MP taken under a stereomicroscope. In the spectra, the blue lines represent the reference spectra from the FTIR library, and the red lines indicate the matched spectra of the identified MPs.

MPs items were identified using ATR-FTIR. Different types of polymers were identified with the dominancy of polyethylene terephthalate (34.2 ± 8.26) followed by polyphenylene sulfide (8.0 ± 4.0), and polyester (4.0 ± 0.71). During polymer analysis, 86.06 ± 18.7 of suspected particles were confirmed as plastics, while 13.94 ± 2.4 consisted of other materials including polyisobutylene, fiberglass, cellulose, which are classified as non-plastics (Fig 1f, S4 Table). No significant differences were observed in the number of different polymer types among the specimens (t = −1.781, n = 5, *p = 0.173*). The images and resultant spectra of the most frequently identified MP items are presented in Fig 2.

### Polymer risk assessment of MPs

The stomach contents of carcasses revealed the presence of small fish, fish bones, and crustaceans including crabs, lobsters, squids, and shrimps. Future detailed dietary analysis at species level will be conducted to better understand the food preferences of IRD. This study used the hazard scores and hazard levels as described by Lithner et al. [60] and Xu et al. [61] to assess the polymer risk of MPs to IRDs (S5 and S6 Tables). The results of the risk assessment of MP polymers depicted that all sampled individuals were present in the range of hazard levels III and IV (medium to high-risk category) in the polymer risk index (H). IRD02 and IRD03 face the medium level (III) MPs hazard risk whereas IRD01, IRD04, and IRD05 are ranked in high risk (IV) category (Table 4).

## Discussion

### Abundance of MPs in the Indus River dolphins

This study represents the first eco-toxicological evaluation of MPs in IRDs providing characterization of MP in this endangered freshwater species. The Indus River is facing high MPs contamination due to industrial expansion, agricultural interventions, unplanned urbanization, and dilated human settlements along the riverbanks as prevalence of MPs reflected by the IRDs. Previous studies have detected MPs in marine cetaceans, but all reported lower quantities compared to the current freshwater study (Table 5). This study detected 184–429 MPs/individual IRD while Battaglia et al. [62] detected 123–422 MPs/individual in bottlenose dolphins *(Tursiops truncatus*) which was slightly lower than our study. Zhang et al. [63] identified the MPs in Indo-Pacific humpback dolphins ranging from 11 to 145 items/individual, while Lusher et al. [52] investigated the gut contents of 21 different cetaceans including two bottlenose dolphins and detected plastic particles ranging from 1 to 88 MPs per individual. Moore et al. [64] conducted the first assessment of MP intake in beluga whales (*Delphinapterus leucas*) and determined the abundance of MPs per individual ranged from 18 to 147 by analyzing samples from stomach, intestine, and feces. These findings align with high MP concentrations previously reported in the Swat and Ravi Rivers, confirming the Indus River as a heavily contaminated freshwater ecosystem with bioaccumulation potential across trophic levels [29,30]. The present study confirmed the presence of high abundance MPs in GIT of all sampled individuals of IRDs with levels exceeding those reported in previous studies. Methodological differences between studies may account for some of the observed variations in MP counts.

**Table 5:** Comparison of the MP abundance in different cetacean species across various regions worldwide.

Species name	No. of samples	Location	Organ	MP items per sample	Reference
Indus River dolphin (*P. minor*)	5	Indus River, Pakistan	Complete GIT	286.4 ± 109.1	Current study
Guiana dolphin (*S. guianesis*)	40	Northwestern Brazil (SW tropical Atlantic)	Stomach	7.77 ± 1.25	Pereira et al. [69]
Common dolphin (*D. delphis*)	15	New Zealand waters	Stomach	184 ± 29	Stockin et al. [11]
Habour porpoises (*P. phocoena*)	30	Germany	Intestine (NS, BS)	18.27 ± 14.54, 8.2 ± 7.89	Philipp et al. [70]
Bottlenose dolphin (*T. truncatus*)	7	Charleston, South Carolina	Stomach, subsample of intestine	280.6 ± 113.0	Battaglia et al. [62]
Indo-pacific humpback dolphin (*S. chinesis*)	12	Peral River Estuary, China	Stomach	53 ± 35.2	Zhang et al. [63]
Striped dolphin (*S. coeruleoalba*)	43	Western Mediterranean Sea	Stomach, intestine	14.9 ± 22.3	Novillo et al. [71]
Habour porpoises (*P. phocoena*)	8	England	Intestine	6.13 ± 2.89	Nelms et al. [72]
Beluga whale	7	Hendrickson Island, Canada	Stomach, intestine, and feces	97 ± 42	Moore et al. [64]
Indo-pacific humpback dolphin (*S. chinesis*)	2	Beibu Gulf, China	Intestine	37.5 ± 7.5	Zhu et al. [7]
Common dolphin (*D. delphis*)	8	South-west England	Intestine	3 ± 3.16	Nelms et al. [73]
Pygmy sperm whale	1	Scotland	Intestine	4	Nelms et al. [72]
East Asina finless porpoises (*N. a. sunameri*)	7	Yellow sea and Bohai sea, China	Intestine	19.1 ± 7.2	Xiong et al. [66]
Common dolphin (*D. delphis*)	35	Galician coast	Stomach	12 ± 8	Hernandez-Gonzalez et al. [67]
Cuvier’s beaked whale	1	Irish waters	Stomach and intestine	≥1	Lusher at al. [52]
Humpback whale	1	Netherlands	Intestine	≤160	Besseling et al. [74]
True’s beaked whale	1	North and west coast of Ireland	Intestine	59	Lusher et al. [50]

Previous studies reported on dolphin species showing MPs observed separately in the stomach, intestinal tracts or sub-sections of the intestines [7,62,65,66]. This study analyzed the complete GIT of IRD rather than random samples, reporting a range of 103–193 MPs per individual in stomach, higher than 3–41 MPs in common dolphins (*Delphis delphis*) [67] but lower than the range of 67–304 MPs per individual in bottlenose dolphins [62]. The range of MPs in the intestinal tract of the IRD is 70–236 items per individual which is greater than found for bottlenose dolphins (45–134 MPs per individual) [62], East Asian finless porpoises (10–32 MPs per individual) [66], and subsamples of the intestines of humpback dolphins (2–45 MPs per individual) [7] (Table 5). The variations in the number of observed MPs are likely due to the type of sample (stomach/intestine) as well as the applied techniques to extract MPs. The observed differences may be influenced by the prevalence of MPs in the research area and the feeding behavior of target species [64,68]. These findings show that the presence of MPs in the intestine of the IRD is higher than in the stomach, attributed to the length and structure of the intestine. Furthermore, this study exhibited a high abundance of MPs as compared to other studied cetaceans.

MPs were randomly dispersed in all stranded carcasses of IRDs, irrespective of age, body length, and sexual maturity. Previous studies have confirmed that body length and maturity did not significantly correlate with the number of MPs in finless porpoises (*Neophocaena*) and harbor porpoises (*Phocoena phocoena*) [22,70]. Similarly, no significant relationship was observed between body length and the number of MPs in humpback dolphins [63]. Battaglia et al. [62] also found no correlation between the total length and MPs abundance in the GIT of stranded bottlenose dolphins. Additionally, Hernandez-Gonzalez et al. [67] discovered no correlation between the total length and the MPs present in the stomach contents of stranded common dolphins. Furthermore, research has also shown no significant impact from sex of dolphins on the accumulation of MPs [63,71]. Thus, all these studies are consistent with the results of the current study suggesting that there is no relation between MPs uptake with body length, maturity, and sex of dolphins.

### Comparison of MPs morphology, size, and its composition

MPs found in the present study within the GIT of stranded IRDs possessed five different morphologies: fibers, films/sheets, fragments, beads, and foams. Fibers were found to be the most abundant (94.76% of MPs), which is consistent with a previous study reported by Lusher et al. [52] that fibers (83.6%) were the most prevalent in cetaceans whereas the remaining items were categorized as fragments (16.4%). Similarly, the abundance of fibers identified in the GIT of humpback dolphins, East Asian finless porpoises, and bottlenose dolphins were comprised of 70.3%, 70%, and 76.1%, respectively [7,64,66]. Moreover, Hernandez-Gonzalez et al. [67] also retrieved 96.6% of fibers from the stomachs of common dolphins which is comparable to the current study. Fibers mainly originate from textile industry and municipal wastewater pollution [75,76]; with a single piece of clothing could generate more than 1900 fibers per wash [77]. Fibers and fragments dominate MPs contamination in aquatic species [78], being consistent with our findings in the Indus River. Aslam et al. [30] demonstrated that MP pollution in the rivers of Pakistan is primarily caused by the direct discharge of wastewater through drains. Our findings indicate that the IRD is significantly affected by the same pollution sources.

In our study, plenty of the MPs were detected in the sieve size of 5 mm-300µm indicating most of the plastic items are micro-sized in IRDs. Our findings indicating the abundance of MPs detected in 5 mm-300µm size, are consistent with the previous studies on humpback dolphins [7], Guiana dolphins, and common dolphins reported that most of the analyzed MPs were observed in size less than 1 mm [69,73]. Subsequently, inferring that a sieve size of 5 mm-300µm is more significant compared to 300µm-150µm and 150µm-50µm.

While characterizing polymers, the presence of PET polymers was most abundant (58.16%) in the IRD being consistent with the PET quantification (39.5%) reported by Aierken et al. [79] and notably higher than the 10.2% reported by Nelms et al. [73]. Potential sources of PET include clothes, food and beverage packaging, furniture, kitchen utensils, and cosmetics [80,81]. The occurrence of PPS was the second highest at 13.61%. The sources of PPS contamination may be untreated wastewater from the electronics and automobile industry [12,63,82]. Similarly, this study found the prevalence of PES, polyvinyl chloride (PVC), polyurethane (PU), and polyethylene (PE) MPs were 6.81%, 2.38%, 2.38%, 1.02% respectively, which correlates the study reported by Nelms et al. [73]. Furthermore, we also found traces of PP, PVA, and PA in the form of fibers, fragments, films, or beads. Polymers emanate from plastic ropes, fishing nets, plastic bottles and bags, and agricultural runoff suggesting their accumulation by intensive anthropogenic interventions [83–86]. The detection of identical polymers in both prey fishes and IRD guts suggests trophic transfer and indicates that local prey species are primary vectors for IRD exposure [29,32]. MPs are transferred up the food chain, and IRDs, as apex predators, accumulate them along with additives including bisphenols and phthalates which are proven to be endocrine disrupting chemicals [71,87,88]. Frequent ingestion of PET, PVC, PE, and related polymers may cause digestive dysfunction, oxidative stress, immune disruption, and reproductive toxicity, collectively threatening the health, survival, and resilience of IRD populations [89,90].

### Risk assessment and hazardous profile of MPs

The analysis of stomach contents revealed dietary preferences of IRDs and the subsequent flow of MP toxicity across trophic levels. However, research on MP toxicity in species commonly found in the IRDs’ diet remains scarce. Future dietary studies focusing on the IRDs’ primary prey species will provide a more comprehensive understanding of the trophic transfer of MPs. The risk assessment of MP polymers specific to cetaceans is likewise scarce, as the majority of research addresses MP prevalence, ingestion rates, and related concerns instead of comprehensive polymer risk assessment. Despite direct assessments on cetaceans are rare, researchers have utilized methods such as the polymer risk index (H) to estimate ecological risks in other aquatic organisms, including fish [56,91], mussel [92], and crabs [93]. By using the same polymer risk index (H), this study highlights the ecological threats posed by MP polymers in IRDs. A non-negligible MP polymer risk was found in IRDs with high hazard scores (Table 4).

This study revealed a medium risk index (Level III) for IRD02 and IRD03, while IRD01, IRD04, and IRD05 were classified under a high-risk category (Level IV) based on hazard score level [60]. The reason for these results might be associated with the location of the respective samples. For instance, IRD04 and IRD05 were found downstream of the Chashma and Taunsa barrages, respectively, areas characterized by substantial pollution loads due to downward water movement, agricultural runoff, untreated sewage and toxic effluents from industries [94,95]. Similarly, IRD01 was also found near the densely populated area of Dera Ghazi Khan main city, facing significant anthropogenic pressure. In contrast, IRD02 and IRD03 were found upstream of Chashma barrage, exhibited a comparatively medium risk (Level III). To date, no comprehensive MPs research has been conducted in the Indus River. However, the identification of high-risk levels of MPs in IRDs emphasizes the high priority needs for comprehensive research to ascertain the prevalence, potential sources and ecological consequences of MPs in the habitat of this species. Such studies are crucial for the effective future conservation of this endangered species.

### Conservation implications and future directions

The extent to which cetaceans are at greater risk of MP exposure, or whether certain cetacean species are more susceptible to MPs ingestion, remains unclear due to a lack of sufficient data [96]. This highlights the need for future studies to gather precise data as evidence of the harmful impacts of MPs on freshwater dolphins including the IRDs. As concluded in our study, the conservation of the IRD faces significant challenges due to the prevalence of MPs in the habitat. We found the highest abundance of MPs compared with other cetaceans, proposing that the high level of MP contamination eventually leads to consequential health risks to the endangered IRDs, because of agricultural runoff, unplanned urbanization, industrial effluents, and bioaccumulation of organic pollutants in Indus River may continue to increase [94,97]. Our ecotoxicological study provides baseline insight into the impacts of MPs on the freshwater ecosystem suggesting the policymakers and legislative bodies to take priority actions for systematic urban planning, sustainable fishing practices, industrial sewage, and pesticidal effluents management for the conservation of IRD. Moreover, local community involvement and awareness campaigns are essential to understanding the adverse impacts of MPs accumulation in rivers, which subsequently affect IRD and integrated biodiversity. Future prospective implications may include more systematic sampling of geographically separated populations, precise ecological data collection, assessment of water and sediment quality of the Indus River. Furthermore, studies on the adverse effects of MPs on the physio-pathological parameters of IRD including the endocrine and digestive systems, liver metabolism, and homeostasis, are essential for conservation efforts.

## Conclusion

This study presents the first evidence of MPs contamination in the GIT of the endangered IRDs, highlighting significant ecological risks to freshwater ecosystem. MPs were detected in all individuals and across all GIT segments, with fibers being the dominant shape and transparent and blue particles were the most common. Most MPs ranged from 5 mm to 300 µm in size, with PET as the predominant polymer. The small intestine contained the highest MP burden, likely due to its anatomical structure. Polymer hazard assessment revealed medium to high risk levels, providing a baseline for evaluating MP impacts in freshwater cetaceans. These findings highlight the urgent need of reducing plastic pollution in freshwater habitats through targeted conservation, monitoring, and management strategies. As a sentinel species, the IRD offers valuable insight into ecosystem health, and further ecotoxicological research is vital to assess long-term impacts of MPs on their survival and reproduction.

## Supporting information

S1 Table Shape-wise distribution of microplastics in Indus River dolphins in this study. (DOCX)

S2 Table Compartment-wise distribution of microplastics from Indus River dolphins. (DOCX)

S3 Table Color-wise distribution of MPs in this study. (DOCX)

S4 Table Composition wise distribution of MPs in this study. (DOCX)

S5 Table Information for the hazard scores and hazard levels of polymer types in this study. (DOCX)

S6 Table Ecological risk assessment of MP polymers. (DOCX)

S1 Checklist Inclusivity in global research. (DOCX)
