# Supplementary material for: First characterization and risk assessment of microplastics in the endangered Indus River dolphin (Platanista minor): Implications for conservation strategies
Source: PLoS One. 2025 Sep 24;20(9):e0330253. doi: 10.1371/journal.pone.0330253 (PMC12459785; doi:10.1371/journal.pone.0330253)
Supplement: S1 Table — (DOCX) [file pone.0330253.s001.docx]

**S1 Table.** Shape-wise distribution of microplastics in Indus River dolphins in this study

| Sample ID | Fibers | Beads | Sheets | Fragments | Foam |
| --- | --- | --- | --- | --- | --- |
| IRD01 | 344 | 2 | 18 | 13 | 0 |
| IRD02 | 413 | 8 | 3 | 4 | 1 |
| IRD03 | 179 | 0 | 2 | 3 | 0 |
| IRD04 | 208 | 1 | 8 | 2 | 0 |
| IRD05 | 213 | 0 | 2 | 8 | 0 |
| Total | 1357 | 11 | 33 | 30 | 1 |
| Mean | 271.4 | 2.2 | 6.6 | 6.0 | 0.2 |
| SD | 101.6 | 3.35 | 6.84 | 4.53 | 0.45 |
| % MP | 94.76 | 0.77 | 2.30 | 2.09 | 0.07 |

Note: SD stands for standard deviation
