# Supplementary material for: First characterization and risk assessment of microplastics in the endangered Indus River dolphin (Platanista minor): Implications for conservation strategies
Source: PLoS One. 2025 Sep 24;20(9):e0330253. doi: 10.1371/journal.pone.0330253 (PMC12459785; doi:10.1371/journal.pone.0330253)
Supplement: S2 Table — (DOCX) [file pone.0330253.s002.docx]

**S2 Table.** Compartment-wise distribution of microplastics from Indus River dolphins

| Sample ID | Esophagus+Fundic Stomach | Main Stomach+Pyloric Chamber | Small Intestine | Large Intestine | Total | Mean | SD |
| --- | --- | --- | --- | --- | --- | --- | --- |
| IRD01 | 81 | 98 | 102 | 96 | 377 | 94.3 | 9.2 |
| IRD02 | 89 | 104 | 174 | 62 | 429 | 107.3 | 47.8 |
| IRD03 | 64 | 50 | 48 | 22 | 184 | 46 | 17.5 |
| IRD04 | 32 | 71 | 57 | 59 | 219 | 54.8 | 16.4 |
| IRD05 | 48 | 64 | 62 | 49 | 223 | 55.8 | 8.4 |
| Total | 314 | 387 | 443 | 288 | 1432 | 286.4 | 109.1 |
| % MPs | 21.93 | 27.03 | 30.94 | 20.11 | 100 |  |  |
