# Supplementary material for: First characterization and risk assessment of microplastics in the endangered Indus River dolphin (Platanista minor): Implications for conservation strategies
Source: PLoS One. 2025 Sep 24;20(9):e0330253. doi: 10.1371/journal.pone.0330253 (PMC12459785; doi:10.1371/journal.pone.0330253)
Supplement: S3 Table — (DOCX) [file pone.0330253.s003.docx]

| Sample ID | Transparent/clear | White | Blue | Red | Green | Pink | Purple | Black/grey | Brown | Yellow | Orange | Total/sample |
| --- | --- | --- | --- | --- | --- | --- | --- | --- | --- | --- | --- | --- |
| IRD01 | 132 | 0 | 98 | 1 | 15 | 53 | 33 | 14 | 4 | 27 | 0 | 377 |
| IRD02 | 190 | 3 | 106 | 2 | 8 | 40 | 47 | 3 | 0 | 30 | 0 | 429 |
| IRD03 | 91 | 0 | 53 | 0 | 3 | 24 | 5 | 1 | 0 | 7 | 0 | 184 |
| IRD04 | 65 | 0 | 87 | 1 | 8 | 21 | 20 | 8 | 0 | 8 | 1 | 219 |
| IRD05 | 74 | 1 | 77 | 0 | 5 | 25 | 19 | 6 | 1 | 15 | 0 | 223 |
| Total | 552 | 4 | 421 | 4 | 39 | 163 | 124 | 32 | 5 | 87 | 1 | 1432 |
| Mean | 110.4 | 0.8 | 84.2 | 0.8 | 7.8 | 32.6 | 24.8 | 6.4 | 1.0 | 17.4 | 0.2 | 286.4 |
| SD | 51.4 | 1.3 | 20.6 | 0.8 | 4.5 | 13.6 | 15.9 | 5.0 | 1.7 | 10.6 | 0.4 | 109.1 |
| % MPs | 38.5 | 0.3 | 29.4 | 0.3 | 2.7 | 11.4 | 8.7 | 2.2 | 0.3 | 6.1 | 0.1 | 100.0 |

**S3 Table.** Color-wise distribution of MPs in this study
