# Supplementary material for: First characterization and risk assessment of microplastics in the endangered Indus River dolphin (Platanista minor): Implications for conservation strategies
Source: PLoS One. 2025 Sep 24;20(9):e0330253. doi: 10.1371/journal.pone.0330253 (PMC12459785; doi:10.1371/journal.pone.0330253)
Supplement: S4 Table — (DOCX) [file pone.0330253.s004.docx]

**S4 Table.** Composition wise distribution of MPs in this study

| Sample ID | | PET | PPS | PES | PVC | PU | PE | PA | PVA | PP | Others | Total |
| --- | --- | --- | --- | --- | --- | --- | --- | --- | --- | --- | --- | --- |
| IRD01 | 34 | | 7 | 5 | 1 | 4 | 0 | 0 | 0 | 0 | 4 | 55 |
| IRD02 | 46 | | 13 | 4 | 0 | 0 | 3 | 1 | 1 | 1 | 25 | 94 |
| IRD03 | 26 | | 3 | 3 | 0 | 1 | 0 | 1 | 0 | 0 | 6 | 40 |
| IRD04 | 27 | | 6 | 4 | 4 | 0 | 0 | 0 | 1 | 0 | 2 | 44 |
| IRD05 | 38 | | 11 | 4 | 2 | 2 | 0 | 0 | 0 | 0 | 4 | 61 |
| Total | 171 | | 40 | 20 | 7 | 7 | 3 | 2 | 2 | 1 | 41 | 294 |
| Mean | 34.2 | | 8.0 | 4.0 | 1.4 | 1.4 | 0.6 | 0.4 | 0.4 | 0.2 | 8.2 | 58.8 |
| SD | 8.26 | | 4.0 | 0.71 | 1.67 | 1.67 | 1.34 | 0.55 | 0.55 | 0.45 | 9.50 | 21.39 |
| % MPs | 58.16 | | 13.61 | 6.80 | 2.38 | 2.38 | 1.02 | 0.68 | 0.68 | 0.34 | 13.95 | 100.00 |
