# Supplementary material for: First characterization and risk assessment of microplastics in the endangered Indus River dolphin (Platanista minor): Implications for conservation strategies
Source: PLoS One. 2025 Sep 24;20(9):e0330253. doi: 10.1371/journal.pone.0330253 (PMC12459785; doi:10.1371/journal.pone.0330253)
Supplement: S5 Table — (DOCX) [file pone.0330253.s005.docx]

**S5 Table.** Information for the hazard scores and hazard levels of polymer types in this study

| Polymer type | Abbreviation | Primary hazard statements^a,b^ | Hazard score (S_j_)^a^ | | Hazard level^a^ |
| --- | --- | --- | --- | --- | --- |
| Polyethylene terephthalate | PET | Carcinogenic and mutagenic, toxic to aquatic life, may cause cancer | | 4 | II |
| Polyphenylene sulfide | PPS | Toxic for reproduction, toxic to aquatic life and long lasting | | 897 | IV |
| Polyester | PES^c^ | Carcinogenic and mutagenic, toxic to aquatic life, long lasting | | 4 | Ⅱ |
| Polyvinyl chloride | PVC | Toxic for reproduction | | 10,001 | V |
| Polyurethane | PU | Toxic to aquatic life and long-lasting effects, may cause cancer | | 7384 | V |
| Polyethylene | PE | Toxic to aquatic life, long lasting | | 11 | III |

^a^ Lithner et al. [1], ^b^ Fang et al. [2], and Li et al. [3] ^c^PES mainly consists of PET; thus, we adopt the score of PET as that of PES.

**Supplemental references**

1. Lithner D, Larsson A, Dave G. Environmental and health hazard ranking and assessment of plastic polymers based on chemical composition. Sci Total Environ. 2011;409(18):3309-24. doi: 10.1016/J.SCITOTENV.2011.04.038.

2. Fang C, Zheng R, Chen H, Hong F, Lin L, Lin H, et al. Comparison of microplastic contamination in fish and bivalves from two major cities in Fujian province, China and the implications for human health. Aquaculture. 2019;512:734322-. doi: 10.1016/J.AQUACULTURE.2019.734322.

3. Li HX, Shi M, Tian F, Lin L, Liu S, Hou R, et al. Microplastics contamination in bivalves from the Daya Bay: Species variability and spatio-temporal distribution and human health risks. Sci Total Environ. 2022;841:156749-. doi: 10.1016/J.SCITOTENV.2022.156749.
