# Supplementary material for: First characterization and risk assessment of microplastics in the endangered Indus River dolphin (Platanista minor): Implications for conservation strategies
Source: PLoS One. 2025 Sep 24;20(9):e0330253. doi: 10.1371/journal.pone.0330253 (PMC12459785; doi:10.1371/journal.pone.0330253)
Supplement: S6 Table — (DOCX) [file pone.0330253.s006.docx]

**S6 Table.** Ecological risk assessment of MP polymers

| **H^a^** | **Risk level^a^** | **Assessment declaration^a^** |
| --- | --- | --- |
| 0-1 | Ⅰ | Very low |
| 1-10 | Ⅱ | Low |
| 10-100 | Ⅲ | Medium |
| 100-1000 | Ⅳ | High |
| >1000 | Ⅴ | Extremely high |

Note. The color of cells represents the degree of hazards such as level I (green), level II (blue), level III (yellow), level IV (orange) and level (red)

^a^ Lithner et al. [1]

**Supplemental references**

1. Lithner D, Larsson A, Dave G. Environmental and health hazard ranking and assessment of plastic polymers based on chemical composition. Sci Total Environ. 2011;409(18):3309-24. doi: 10.1016/J.SCITOTENV.2011.04.038.
